# Maltol, a Natural Flavor Enhancer, Inhibits NLRP3 and Non-Canonical Inflammasome Activation

**DOI:** 10.3390/antiox11101923

**Published:** 2022-09-28

**Authors:** Huijeong Ahn, Gilyoung Lee, Byung-Cheol Han, Seung-Ho Lee, Geun-Shik Lee

**Affiliations:** 1 College of Veterinary Medicine and Institute of Veterinary Science, Kangwon National University, Chuncheon 24341, Korea; 2 Korea Ginseng Research Institute, Korea Ginseng Corporation, Daejeon 34337, Korea

**Keywords:** maltol, inflammasome, macrophages, interleukin-1beta

## Abstract

Maltol (3-hydroxy-2-methyl-4-pyrone) is used widely as a food and cosmetic supplement, and it has antioxidant and anti-inflammatory activities. Inflammasome causes the maturation and secretion of interleukin (IL)-1β and -18 through the activation of caspase-1 (Casp1), which contributes to various inflammatory diseases. This study examined the effects of maltol on the inflammasome activation in macrophages and mice. Lipopolysaccharide (LPS)-primed macrophages were treated with a trigger of NLRP3, NLRC4, AIM2, or non-canonical (NC) inflammasomes in the presence of maltol. The secretion of IL-1β and IL-18 and the cleavage of Casp1 were analyzed as indices of inflammasome activation. Mice were injected with LPS and an NLRP3 trigger with or without maltol, and the peritoneal IL-1β secretions were observed. The effects of maltol on reactive oxygen species (ROS) production and Casp1 activity were analyzed to determine the mechanism. Maltol inhibited the activation of NLRP3 and NC inflammasomes, but it did not alter the other inflammasomes. Maltol also attenuated IL-1β secretion resulting from the inflammasome activation in mice. The anti-inflammatory mechanism of maltol was revealed by the inhibition of ROS production and Casp1 activity. Maltol is suggested to be promising as a anti-inflammasome molecule.

## 1. Introduction

Inflammasome, an intracellular multi-protein complex in innate immune cells and some epithelial cells, initiates and amplifies inflammation through the maturation and secretion of inflammatory caspases and pro-inflammatory cytokines [1]. Inflammasome consists of (a) a sensor protein (e.g., nucleotide-binding oligomerization domain, leucine-rich repeat, and pyrin domain containing 3 [NLRP3]; nod-like receptor family caspase recruitment domain containing protein 4 [NLRC4]; absent in melanoma 2 [AIM2]), (b) an effector protein (caspase-1 [Casp1]), and (c) an adaptor protein (apoptosis-associated speck-like protein containing a caspase recruitment domain [ASC]) [2]. An NLRP3 inflammasome is formed when the cell is engaged with a danger molecule, such as adenosine phosphate (ATP), a bacterial toxin (e.g., nigericin), and monosodium urate crystals [2]. NLRP3 triggers induce the assembly of an inflammasome through common intracellular signaling, such as potassium efflux and mitochondrial reactive oxygen species (ROS) production [2,3]. Otherwise, NLRC4 and AIM2 inflammasomes are assembled when the sensor protein is in contact with specific triggers in the cytoplasm, such as the flagellin and dsDNA [4]. In general, the lipopolysaccharide (LPS) interacts with toll-like receptor (TLR) 4 on the plasma membrane, leading to an inflammatory response through nuclear factor (NF)-κB signaling, but cytosolic LPS is sensed directly by caspase-11 (caspase-4/5 in humans), triggering inflammasome signaling, which is called the non-canonical (NC) inflammasome [4,5]. The activated Casp1 resulting from inflammasome activation proteolytically cleaves the pro-form of cytokines (e.g., interleukin [IL]-1β, and IL-18) and gasdermin D (GSDMD), and induces inflammatory cell death (i.e., pyroptosis) [2]. The cleaved GSDMD forms a pore on the cell membrane, facilitating the release of the cytosolic contents, such as IL-1β and lactate dehydrogenase (LDH) [2,4]. An inflammasome is activated by two signals: priming and activation steps [1]. The TLR4/NF-κB signaling upregulates the inflammasome components (e.g., NLRP3 protein and pro-IL-1β) during the priming step and the inflammasome trigger then induces the assembly of the inflammasome in the activation step [1,6]. Inflammasomes are a key axis of the pathogenesis of metabolic and degenerative diseases, such as Alzheimer’s disease, type 2 diabetes, neurological disorders, and liver and kidney diseases [2,3,4,7,8]. Thus, the screening for anti-inflammasome molecules has attracted considerable interest [2,9,10]. 

**Figure 1:**
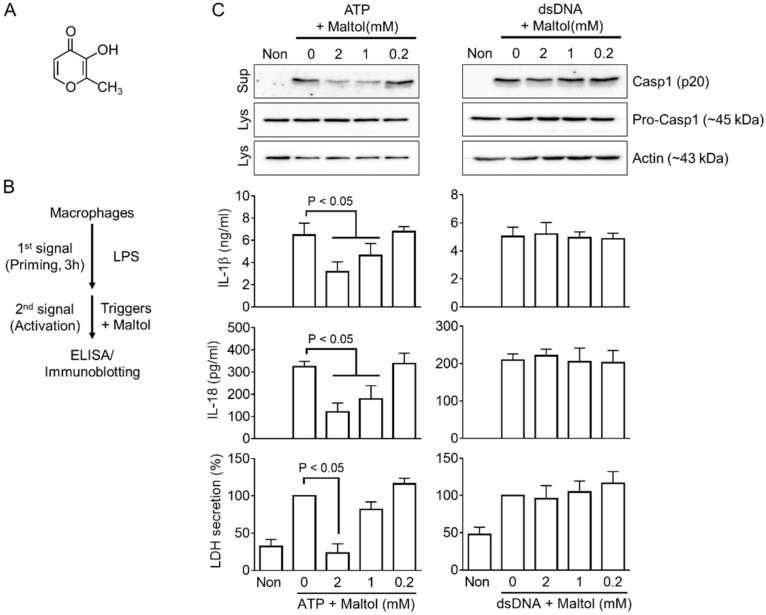
Effects of maltol on NLRP3 and AIM2 inflammasome activation. (**A**), Chemical structure of maltol (C_6_H_6_O_3_). (**B**), Schematic diagram describing the mode of inflammasome activation. Macrophages were primed with LPS for 3 h and treated with an inflammasome trigger with maltol. The indices of inflammasome activation were analyzed by ELISA and Western blotting. (**C**), LPS-primed BMDMs were induced to activate NLRP3 or AIM2 inflammasome with ATP or dsDNA in the presence of maltol, as indicated. The maturation of secretion of Casp1 (p20), IL-1β, IL-18, and LDH was analyzed by immunoblotting or ELISA. All immunoblot data shown are representative of at least two independent experiments. The bar graph presents the mean ± SD.

Maltol (3-hydroxy-2-methyl-4-pyrone, Figure 1A), a natural organic compound, has the odor of cotton candy and caramel, and it is used widely as a flavor enhancer in the food, cosmetics, and pharmaceutical industries [11]. Maltol is found in roasted malt and is synthesized from maltose and amino acids by the Maillard reaction during the heat processing of Korean red ginseng (KRG) [12]. Maltol has been in the spotlight as a molecule exhibiting the anti-inflammatory efficacy of KRG, a well-known herbal medicine. It has been consumed to ameliorate several human health concerns for a long time in Northeast Asia, including Korea and China [11,13]. The contents of maltol increase during the heat processing of KRG. The free radical scavenging activity is also increased due to the increasing maltol content [14]. Maltol is a potent antioxidant that has been reported to ameliorate several diseases of the nervous system [15,16,17,18], liver [19,20,21], kidney [22], and diabetes [18]. Maltol is a major and effective ingredient of the non-saponin fractions of ginsengs [11,23]. KRG inhibits or stimulates inflammasome activation depending on its composition [2,9,10]. 

Thus, this study tested the effects of maltol on inflammasome activation. Human and murine macrophages were activated by several inflammasomes using a selective trigger of NLRP3, NLRC4, AIM2, and NC inflammasomes in the presence of maltol. In addition, the anti-inflammasome property of maltol was elucidated in an animal model. Mechanistic studies of maltol as an anti-inflammasome molecule were conducted.

## 2. Materials and Methods

Unless stated otherwise, all culturing ingredients were purchased from Welgene Inc. (Gyeongsan-si, Gyeongsanbuk-do, Korea). All plastics were obtained from SPL Life Sciences (Pocheon-si, Gyeonggi-do, Korea), and the chemicals were supplied by Daejung Chemicals & Metals Co., Ltd. (Siheung-si, Gyeonggi-do, Korea). 

### 2.1. Preparation of Macrophages

For bone marrow-derived macrophages (BMDMs), the bone marrow progenitor cells were isolated from the tibia and femur of mice (C57BL/6, 6- to 12-week-old, Narabio Co., Seoul, Korea) [9]. The cells were incubated with DMEM media containing 10% fetal bovine serum (FBS, S 001-01, Welgene Inc., Gyeongsan-si, Korea), 50% of L929 cell-conditioned medium as a source of macrophage colony-stimulating factor, and antibiotics at 37 °C in a 5% CO_2_ atmosphere for seven days. THP-1 cells (40202, Seoul, Korea, Korean Cell Line Bank), human monocyte-like cells, were maintained in RPMI 1640 media containing 10% FBS and antibiotics [5]. These cells were differentiated into macrophage-like cells by a treatment of 200 nM phorbol 12-myristate 13-acetate (PMA, InvivoGen, San Diego, CA, USA) for 24 h. 

### 2.2. Treatment to Activate Inflammasome

As shown in Figure 1B, macrophages (i.e., BMDMs and PMA-treated THP-1 cells) were seeded on a 12-well plate (1.0 × 10^6^ cells per well) in RPMI 1640 containing 10% FBS and antibiotics, and treated with LPS (1 μg/mL; L4130, Sigma-Aldrich Co., Burlington, MA, USA) for 3 h [5,9,10]. The LPS-primed cells were replaced with RPMI 1640 media (350 μL per well) containing an inflammasome trigger with maltol (ESFOOD, #186785643, Gunpo-si, Gyeonggi-do, Korea). The inflammasome triggers are as follows: ATP (5 mM, InvivoGen, San Diego, CA, USA) for 1 h, nigericin (NG, 40 μM, Tocris Bioscience, Bristol, UK) for 1 h, monosodium urate crystals (MSU, 400 μg/mL, Sigma-Aldrich Co., Burlington, MA, USA) for 3 h, flagellin (500 ng/mL; Invivogen, San Diego, CA, USA) with Lipofectamine 2000 (10 μL/mL, Invitrogen, Carlsbad, CA, USA) for 3 h, dsDNA (1 μg/mL) with jetPRIME^TM^ (2 μL/mL, Polyplus-transfection Inc., Illkirch, France) for 1 h, LPS (2 μg/mL, Sigma-Aldrich Co., Burlington, MA, USA) with FuGENE^®^ HD (2.5 μL/mL, Roche, Penzberg, Germany) for 6 h, and rotenone (160 μM, Santa Cruz Biotechnology, Dallas, TX, USA) for 6 h [24,25].

### 2.3. Animal Experiment

C57BL/6 female mice (seven weeks old, Narabio Co., Gunsan, Korea) were acclimated to the environment for one week under a 12 h light/dark cycle at 18 to 24 °C. The mice were provided a normal chow diet and tap water ad libitum. The mice were injected intraperitoneally (IP) with LPS (5 mg/kg) in 200 μL phosphate-buffered saline (PBS). After 5 h LPS injection, the mice were IP administered with NG (3 mg/kg) and maltol (100 mg/kg) in 200 μL PBS [26]. In addition, the other groups of mice were fed daily with maltol (100 mg/kg) for seven days and then injected with LPS (5 mg/kg) and NG (3 mg/kg) After an additional 1 h, the mice were euthanized by CO_2_ inhalation and cervical dislocation, and the peritoneal lavages were collected. PBS (5 mL) was administered into the peritoneal cavity and shaken gently. The lavages (>4 mL) were then collected for the assays. All animal experiments were carried out in accordance with the National Institutes of Health Guide for the Care and Use of Laboratory Animals and approved by the Institutional Animal Care and Use Committee of Kangwon National University (IACUC; approval no. KW-220401-4 and KW-220405-1).

### 2.4. Western Blot Analysis

After inflammasome triggering, the cellular supernatants (Sup) were collected for the assays. The cell lysate (Lys) was prepared with a lysis buffer containing 0.01% Triton X-100, NaCl (150 mM), Tris-base (50 mM, pH 8.0), and protease inhibitors (HaltTM cocktail, ThermoFisher Scientific, Waltham, MA, USA), and then harvested by centrifugation. The Sup and Lys were electrophoresed by 10 or 16% SDS-PAGE and transferred to PVDF membranes (GE Healthcare Bio-Science, Pittsburgh, PA, USA) [25,27]. The membranes were incubated with the following 1st antibodies: anti-Casp1 (p20) antibody (AG-20B-0042-C100, AdipoGen Co., San Diego, CA, USA), anti-NLRP3 antibody (AG-20B-0014-C100, AdipoGen^®^ Co., San Diego, CA, USA), anti-mouse IL-1β antibody (AF-401-NA, R&D Systems, Minneapolis, MN, USA), or anti-Actin antibody (sc-1615, Santa Cruz Biotechnology, Dallas, TX, USA). After overnight incubation at 4 °C, the membrane was proved with the 2nd antibody conjugated with horseradish peroxidase (Invitrogen, San Diego, CA, USA or Abcam, Cambridge, UK) for 2 h at room temperature. The immunoblotting images were obtained using a chemiluminescent system (EZ-Capture II, ATTO Technology, Tokyo, Japan).

### 2.5. Assays for Cytokines, LDH Secretion, ROS Production, and Casp1 Activity

The IL-1β and IL-18 concentrations were measured using an enzyme-linked immunosorbent assay (ELISA, Helsinki, Finland) kit (DY201, DY7625 or DY401, R&D Systems, Minneapolis, MN, USA) [27]. The sup and the lavages were applied to the anti-sera coated plates, and the reagents and solution proved from the manufacturer were then added. The plates were measured at 450 nm with the reference wavelength of 655 nm using a multi-microplate spectrophotometer (Synergy™ H1 Hybrid Multi-Mode Reader, BioTek, Winooski, VT, USA).

LDH secretion was assayed using a kit according to the manufacturer’s protocol (BCT-LDHP, Biomax, Seoul, Korea). The sup was transferred to a 96-well plate, and the reagent was added to each well and incubated for 30 min [28]. The absorbance signal was measured at 490 nm using a multi-microplate spectrophotometer.

ROS production was measured using dihydrorhodamine 123 (DHR123, Cayman Chemical, Ann Arbor, MI, USA) [29]. The LPS-primed BMDMs (1.25 × 10^5^ cells/well in a 96-well-black plate) were treated with DHR123 and rotenone (160 μM) in the presence of maltol or diphenyleneiodonium (DPI, 200 μM, Tocris Bioscience, Bristol, UK). After incubation for 1 h, the cell cultures were washed twice with PBS, and the fluorescence signal was detected at excitation and emission wavelengths of 500 nm and 536 nm, respectively, using the multi-microplate spectrophotometer.

The Casp1 or caspase-4 (Casp4) activities were assayed using recombinant human Casp1 or Casp4 (one unit per reaction, Biovision, Milpitas, CA, USA) and a Casp1 fluorometric assay kit (Biovision, Zurich, Switzerland) or Ac-LEVD-AMD, a substrate of Casp4, (Enzo Life Sciences, Inc., Farmingdale, NY, USA) in the presence of maltol or the pan-caspase inhibitor (Z-VAD-FMK, 10 μg/mL; R&D System, Minneapolis, MN, USA) [5]. The plates were measured at excitation and emission wavelengths of 400 and 505 nm for Casp1, or 360 and 450 nm for Casp4, using the multi-microplate spectrophotometer.

### 2.6. Statistical Analyses

Statistical analyses were conducted using a Mann–Whitney test for the two groups or Tukey’s multiple comparisons test for multiple groups using GraphPad Prism 6 (GraphPad Software, San Diego, CA, USA). The *p*-value is shown in the figures.

## 3. Results

### 3.1. Maltol Is a Candidate for an Inflammasome Regulator

The effects of maltol (Figure 1A) on the inflammasome activation were assessed by priming bone marrow-derived macrophages (BMDMs) with LPS as a first signal and subjecting them to inflammasome triggers with/without maltol during the activation step (Figure 1B). Maltol (up to 20 mM) showed no cytotoxicity to the BMDMs (Supplementary Figure S1A). The secretions of IL-1β, IL-18, LDH, and Casp1 (p20) were measured as indices of inflammasome activation. As shown in Figure 1C, both NLRP3 and AIM2 inflammasome triggers, ATP and dsDNA, induced the secretion of the indices from LPS-primed BMDMs as expected. On the other hand, the cotreatment of maltol with ATP in the BMDMs attenuated the secretion of Casp1 (p20), IL-1β, IL-18, and LDH, while it did not alter the secretion of dsDNA-transfected BMDMs (Figure 1C). In addition, maltol inhibited the cleavage of gasdermin D (GSDMD) and the formation of an Asc pyroptosome in response to ATP (Supplementary Figure S1B,C). Overall, maltol regulates the inflammasome activation mediated by ATP but not by dsDNA.

### 3.2. Maltol Attenuates the NLRP3 Inflammasome

**Figure 2:**
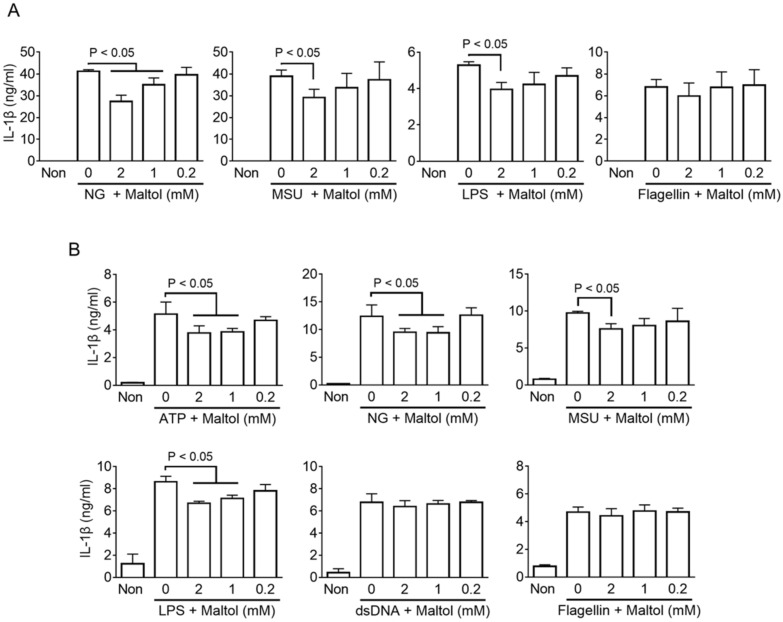
Effect of maltol on various inflammasome activation in murine and human macrophages (**A**), LPS-primed BMDMs were activated NLRP3 (NG and MSU), NC (LPS), or NLRC4 (flagellin) inflammasomes by a specific trigger and co-treated with maltol as indicated. IL-1β secretions were analyzed by ELISA. (**B**), PMA-treated THP-1, human macrophage-like cells, were treated with NLRP3 (ATP, NG, MSU), NC (LPS), AIM2 (dsDNA), or NLRC4 (flagellin) inflammasome triggers to activate the inflammasome in the presence of maltol. IL-1β secretion was measured by ELISA. The bar graph presents the mean ± SD.

The regulating property of maltol on inflammasome activation was confirmed by various inflammasome triggers in human and murine macrophages. LPS-primed BMDMs were treated with NLRP3 triggers (NG and MSU), NC triggers (LPS transfection), or NLRC4 triggers (flagellin transfection) in the presence of maltol, and the secretions of IL-1β, IL-18, and LDH were measured. As a result (Figure 2A and Supplementary Figure S2A), maltol dose-dependently inhibited the release of IL-1β, IL-18, and LDH induced by the NLRP3 and NC inflammasome activations in the murine macrophages, but it did not alter the releases resulting from NLRC4 inflammasome activation. This suggests that maltol acts as a selective inhibitor of NLRP3 inflammasome. Next, the anti-inflammasome properties of maltol were confirmed using PMA-treated THP-1 cells, which are human macrophage-like cells. Maltol significantly attenuated the IL-1β secretion from the LPS-primed THP-1 treated with ATP, NG, and MSU (Figure 2B). In addition, maltol inhibited the IL-1β releases resulting from the LPS transfection. IL-18 and LDH secretions were induced by the ATP and NG treatments, and maltol attenuated the transfection of LPS (Supplementary Figure S2B). On the other hand, maltol did not change the IL-1β secretion followed by the AIM2 and NLRC4 inflammasome activation in human cells. Hence, maltol is a selective blocker of NLRP3 and NC inflammasome signaling.

### 3.3. Maltol Attenuates the Peritoneal IL-1β Production in NLRP3 Inflammasome Activating Mice

**Figure 3:**
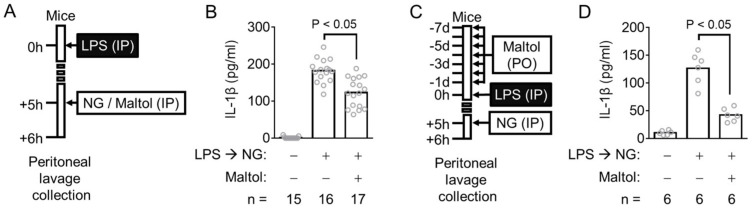
Effect of maltol on the inflammasome activation in mice. (**A**), Schematic diagram describes the mode of the maltol injecting experiment. (**B**), The peritoneal IL-1β production of the mice treated similar to panel A was measured. (**C**), The procedure for oral maltol administration is shown. (**D**), The secretion of peritoneal IL-1β of the maltol-fed animals was analyzed. IL-1β releases were measured by ELISA. The bar graph presents the mean, and each gray circle indicates a value from each mouse.

The anti-inflammasome property of maltol was examined further in a mouse model. The mice were injected intraperitoneally with LPS and then treated with an NLRP3 trigger (NG) with/without maltol (Figure 3A). The IL-1β in the peritoneal cavity was measured as a readout of the NLRP3 inflammasome activation. As expected, peritoneal IL-1β secretion was induced by LPS and NG injection. This IL-1β production was attenuated by the maltol treatment (Figure 3B). In addition, the animals were daily fed with maltol for 7d, and then injected with LPS and NG, as shown in Figure 3C. Peritoneal IL-1β production was inhibited significantly by the oral administration of maltol (Figure 3D). Thus, maltol is suggested as an inhibitor of both in vivo and in vitro NLRP3 signaling.

### 3.4. Maltol Inhibits NLRP3 Inflammasome through Inhibition of ROS Production and Casp1 Activity

The effects of maltol on several signaling molecules that induce inflammasome activation, such as the priming step, ROS production, and Casp1 activity, were examined to determine how maltol disrupts the activation of the NLRP3 inflammasome. The upregulation of the inflammasome components, such as NLRP3 and pro-IL-1β, during the priming step acts as a gatekeeper to assembling the NLRP3 inflammasome and as a substrate of the inflammasome activation [1]. BMDMs were treated with maltol instead of LPS during the priming step, and the levels of NLRP3 and pro-IL-1β expression were measured. NLRP3 and pro-IL-1β were upregulated by maltol, but the levels were relatively lower than LPS (Supplementary Figure S3A,B). Next, this study tested whether maltol induces the priming as a first signal. The maltol-primed BMDMs did not induce IL-1β and Casp1 (p20) secretion after the NLRP3 inflammasome activation, while LPS-primed BMDMs did (Supplementary Figure S3C). In addition, the role of maltol on cytokine production in mice was tested. Maltol did not induce peritoneal IL-1β production, but LPS did (Supplementary Figure S3D). Thus, maltol does not affect the priming step.

**Figure 4:**
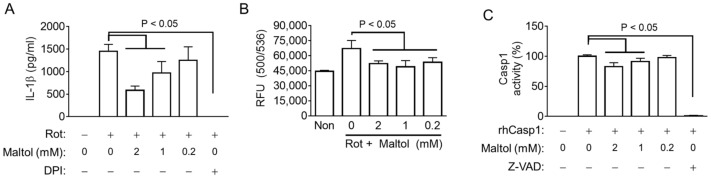
Effect of maltol on the ROS production and Casp1 activity. (**A**), LPS-primed BMDMs were treated with rotenone (Rot) in the presence of maltol or DPI (a ROS scavenger). IL-1β secretion was analyzed by ELISA. (**B**), LPS-primed BMDMs were treated with DHR123 to measure ROS production in the presence of Rot with/without Maltol. Relative fluorescence unit (RFU) as the level of ROS was measured. (**C**), Recombinant human (rh) Casp1 was incubated with maltol or Z-VAD-FMK (Z-VAD, a pan-caspase inhibitor), and the Casp1 activity was analyzed using an assay kit. The bar graph presents the mean ± SD.

Next, the role of maltol on ROS production, which has been reported as a common signaling for NLRP3 inflammasome activation, was elucidated [30]. Rotenone, which increases ROS generation through mitochondria dysregulation, acts as a selective NLRP3 trigger [2,30]. LPS-primed BMDMs were treated with rotenone, leading to IL-1β secretion, which was blocked by maltol and an ROS scavenger (DPI) (Figure 4A). Subsequently, the cellular ROS levels were measured in the LPS-primed BMDM-treated rotenone with/without maltol. As a result (Figure 4B), rotenone increased the intracellular ROS, and maltol significantly blocked ROS production. In addition, ATP and NG induced the level of ROS in the BMDMs, and the maltol co-treatment attenuated the increasing ROS production (Supplementary Figure S4A). Overall, maltol inhibits the activation of the NLRP3 inflammasome by inhibiting ROS production.

This study examined whether maltol changes the activity of Casp1, an effect protein of the inflammasome. Recombinant human Casp1 was reacted with its substrates in the presence of maltol. Maltol attenuated the activity of Casp1 similar to Z-VAD-FMK, a pan-caspase inhibitor (Figure 4C). However, maltol did not change the Casp4 activity (Supplementary Figure S4B). Hence, maltol interrupts NLRP3 signaling by inhibiting ROS production and Casp1 activity.

## 4. Discussion

The pharmacological value of maltol has been studied in various disease models because it has potent antioxidant efficacy. In streptozotocin (STZ)-induced diabetic rats, maltol inhibited the generation of advanced glycation end products (AGEs) and ameliorated diabetic renal toxicity [15]. In the D-galactose-induced aging and tissue injury model, maltol prevented the accumulation of D-galactose-mediated AGEs and liver and kidney injuries in mice [18]. In addition, maltol alleviated the peripheral neuropathy in STZ-treated rats and blocked STZ-induced apoptosis in a Schwann cell line [17]. Moreover, maltol was reported to protect from oxidative stress in the nervous system [16]. That is, maltol increased the viability of the H_2_O_2_-treated retinal ganglion cells and reduced NF-κB phosphorylation [16]. The NLRP3 inflammasome is attracting attention because it contributes to the pathogenesis of metabolic and degeneration diseases, such as diabetes and neurological disorders [3,8]. The therapeutic efficacy of maltol on the symptoms of diabetes and neuroinflammation suggests that maltol regulates NLRP3 inflammasome activation.

The efficacy of maltol on liver inflammation and fibrosis has been reported. The hepatoprotective activity of maltol isolated from KRG was confirmed on alcohol-induced oxidative damage in mice [19]. In this paper, maltol reduced the serum aspartate transaminase (AST) level, the alanine transaminase (ALT) level, tumor necrosis factor (TNF) α and IL-1β production in the liver. In addition, maltol blocked the alcohol-induced hepatocyte apoptosis. Maltol also prevented hepatic dysfunction and oxidative stress injury in acetaminophen-induced hepatotoxicity in mice [21]. In addition, maltol attenuated the NF-κB signaling, resulting in a decrease in serum TNFα and IL-1β [21]. In another study, maltol mitigated thioacetamide-induced liver fibrosis and inhibited thioacetamide-induced ALT, AST, and oxidative stress indices [20]. Liver diseases are induced mainly by inflammation [7]. Thus, inflammasome activation has been studied in human and experimental hepatic disease models because it has been revealed as a major contributor to liver diseases [7]. Based on the current study, the beneficial effects of maltol on liver diseases might be supported by its anti-inflammasome property.

Maltol has been studied progressively on the side effects of cisplatin, an anti-cancer medicine. The cisplatin-treated mice presented nephrotoxicity, which was ameliorated by maltol, and it also reduced the serum TNFα and IL-1β secretion via the reduction in oxidative stress [22]. In addition, maltol ameliorated the cisplatin-induced intestinal toxicity and inhibited cisplatin-mediated apoptosis in the intestinal epithelial cell line [31]. Interestingly, the authors mentioned that maltol inhibited NLRP3 inflammasome activation, but they only observed the protein levels of inflammasome components, such as NLRP3, Casp1, pro-IL-1β, and GSDMD [31]. This suggests that maltol attenuates the expression of the inflammasome components by inhibiting NF-κB signaling [16,21,31]. On the other hand, maltol increased the NLRP3 and pro-IL-1β protein levels in BMDMs, while it could not induce the priming step of inflammasome activation (Supplementary Figure S1D). It suggests that maltol might oppositely regulate NF-κB signaling depending on the cell types. In conclusion, the pharmacological properties of maltol are based on its anti-oxidative and anti-inflammasome efficacy.

## 5. Conclusions

This study examined the effect of maltol on the activation of various inflammasomes in macrophages. Maltol inhibited the activation of NLRP3 and NC inflammasomes in human and murine cells, while it did not alter the assembly of NLRC4 and AIM2 inflammasomes. In addition, maltol attenuated peritoneal IL-1β secretion from the mice injected with LPS and NG, a well-known NLRP3 trigger. Interestingly, maltol partially stimulated the expression of inflammasome components, such as NLRP3 and pro-IL-1β. However, the maltol-primed BMDMs could not activate the NLRP3 inflammasome, and it did not produce any peritoneal IL-1β in mice. Mechanistic studies showed that maltol attenuated ROS generation through mitochondrial dysregulation and blocked the IL-1β secretion in response to mitochondrial ROS, a common signal of NLRP3 inflammasome activation. In addition, maltol inhibited the activity of recombinant Casp1. Overall, maltol is promising as an anti-inflammasome molecule.

## Data Availability

Data is contained within the article.

## Supplementary Materials

The following supporting information can be downloaded at: https://www.mdpi.com/article/10.3390/antiox11101923/s1, Figure S1: Effect of maltol on the cytotoxicity, GSDMD cleavage and ASC pyroptosomes, Figure S2: Effect of maltol on IL-18 and LDH secretion, Figure S3: Effect of maltol on the priming step of inflammasome activation, Figure S4: Effect of maltol on ROS production and Caspase 4 activity.
