# Supplementary material for: Maltol, a Natural Flavor Enhancer, Inhibits NLRP3 and Non-Canonical Inflammasome Activation
Source: Antioxidants (Basel). 2022 Sep 28;11(10):1923. doi: 10.3390/antiox11101923 (PMC9598623; doi:10.3390/antiox11101923)
Supplement: Supplementary file 1 [file antioxidants-11-01923-s001.zip › antioxidants-1927344-supplementary.pdf]

Supplementary Information

Maltol, a natural flavor enhancer, inhibits NLRP3 and non-canonical inflammasome activation

Huijeong Ahn[1], Gilyoung Lee[1], Byung-Cheol Han[1,2], Seung-Ho Lee[2], and Geun-Shik Lee[1,*]

[1]College of Veterinary Medicine and Institute of Veterinary Science, Kangwon National University, Chuncheon, Republic of Korea.

[2]Korea Ginseng Research Institute, Korea Ginseng Corporation, Daejeon, Republic of Korea.

Running title: Anti-inflammasome effect of maltol

*Correspondence: Geun-Shik Lee, DVM, Ph.D.

Lab. of Inflammatory Diseases, Department of Physiology, College of Veterinary Medicine, Kangwon National University, 1 kangwondaehak-gil, Chuncheon 24341, Republic of Korea.
e-mail: leegeun@kangwon.ac.kr, Tel: +82-33-250-8683, Fax: +82-33-244-2367

Supplementary Figure S1

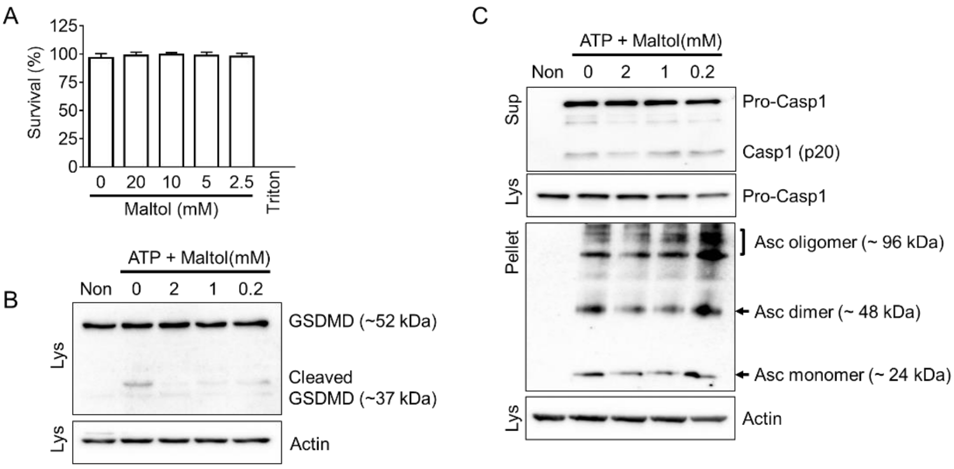

A, LPS-primed BMDMs were treated with maltol or Triton X-100 (0.01%, Triton), and the survival rate (cytotoxicity) was analyzed using an assay kit (EZ-Cytox, Dogen Bio, Seoul, Republic of Korea) according to the manufacture's method. The bar graph presents the mean ± SD. **B**, LPS-primed BMDMs were treated with ATP and maltol as indicated, and the cleavage of GSDMD was observed using immunoblotting (anti-GSDMD antibody, ab209845, Abcam, Cambridge, MA, USA). **C**, LPS-primed BMDMs were treated with ATP and maltol. The lysate (Lys) was transferred into a new tube and collected by centrifugation at 15,000 rcf for 5 min. The remaining pellet was washed two times with PBS and then re-suspended and cross-linked with 2 mM suberic acid bis (Sigma-Aldrich Co.) for 1 h, followed by centrifugation at 15,000 rcf for 5 min. The cross-linked pellets (Pellet) were re-suspended in 50 μL of 2 X loading dye buffer (116 mM Tris, 3.4 % SDS, 12 % glycerol, 200 mM DTT, 0.003 % bromo phenol blue) [Ref.1, 2]. The pellet was subjected to Western blot assay using anti-ASC antibody (sc-22514, Santa Cruz Biotechnology, Dallas, TX, USA).

Ref. 1. Lee, G.S.; Subramanian, N.; Kim, A.I.; Aksentijevich, I.; Goldbach-Mansky, R.; Sacks, D.B.; Germain, R.N.; Kastner, D.L.; Chae, J.J. The calcium-sensing receptor regulates the NLRP3 inflammasome through Ca2+ and cAMP. Nature 2012, 492, 123-127, doi:10.1038/nature11588.

Ref. 2. Fernandes-Alnemri, T.; Yu, J.W.; Juliana, C.; Solorzano, L.; Kang, S.; Wu, J.; Datta, P.; McCormick, M.; Huang, L.; McDermott, E.; et al. The AIM2 inflammasome is critical for innate immunity to Francisella tularensis. Nature immunology 2010, 11, 385-393, doi:10.1038/ni.1859.

Supplementary Figure S2

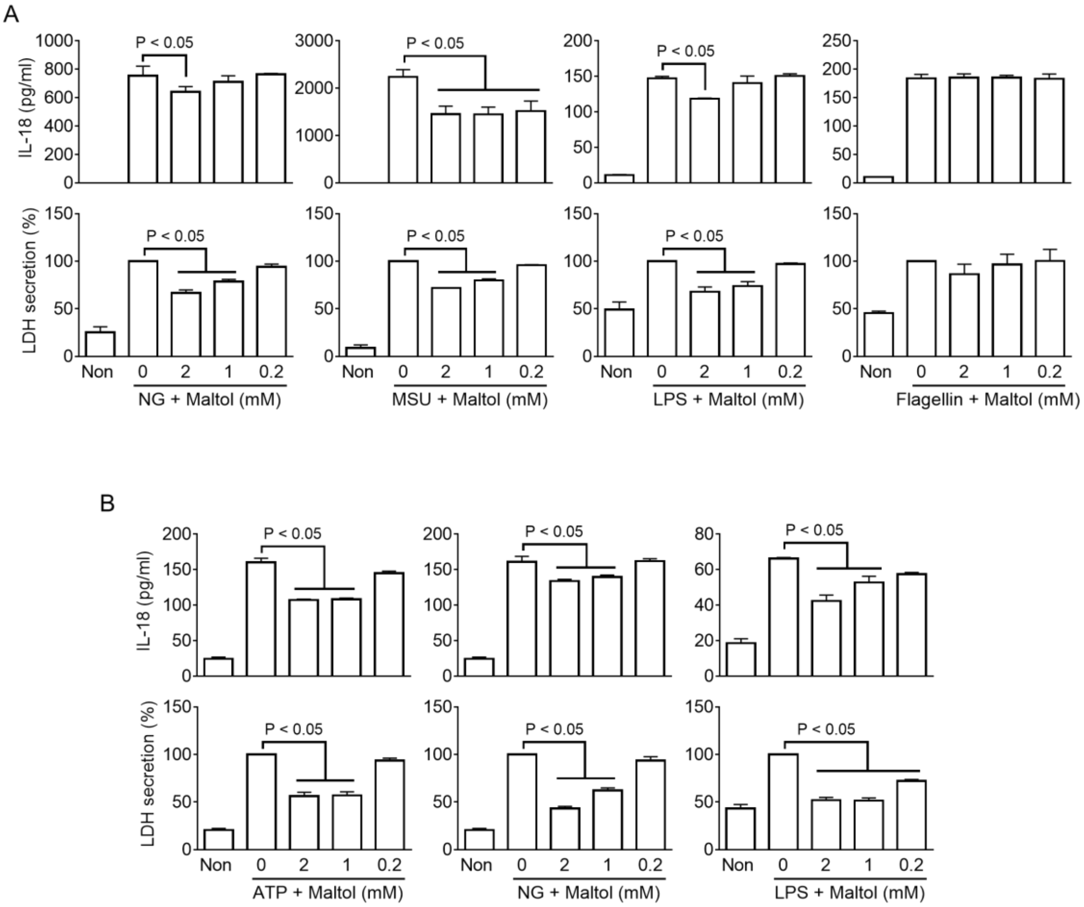

A, LPS-primed BMDMs were treated with the inflammasome trigger, and the secretion of IL-18 and LDH was measured. **B**, PMA-treated THP-1 cells were primed with LPS, and the inflammasomes were activated with the selective triggers. IL-18 and LDH releases were analyzed. The bar graph presents the mean ± SD.

Supplementary Figure S3

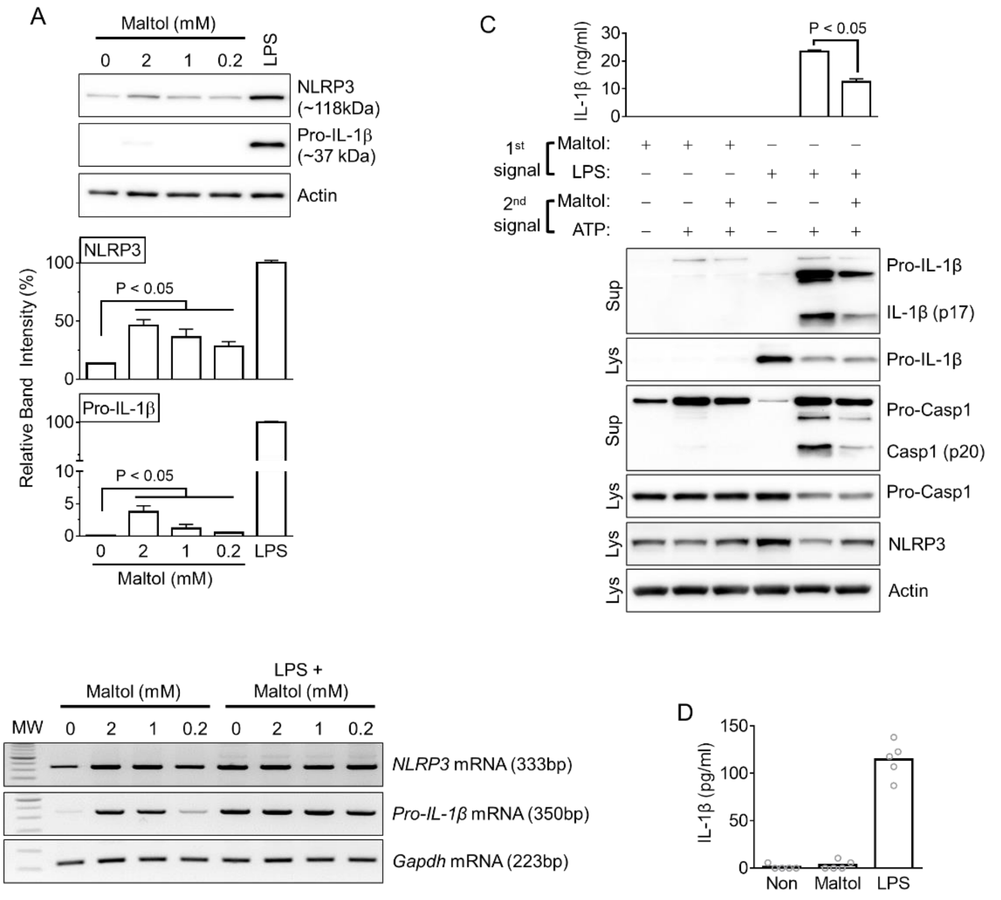

A, BMDMs were treated with maltol or LPS for 3 h, and the lysates were collected for analyzing the protein levels of NLRP3 and pro-IL-1β using immunoblotting. The band intensities of the images are plotted as bar graphs. **B**, BMDMs were treated with maltol only, or LPS and maltol as indicated. The transcripts of *NLRP3* and *pro-IL-1β* were analyzed using RT-PCR. The gene-specific primers are as follows: *NLRP3* (GeneBank ID, NM_145827), 5’-CAG ACT GGC AAA AGG CTG TG-3’, 5’- TCT TCC CGG TCT CCA TCT GT-3’; *pro-IL-1β* (NM_008361), 5’-CAG GCA GGC AGT ATC ACT CA-3’ and 5’-AGG CCA CAG GTA TTT TGT CG-3’; *Gapdh* (NM_001289726, 223 bp), 5’-AAC TTT GGC ATT GTG GAA GG-3’ and 5’-ACA CAT TGG GGG TAG GAA CA-3’. The images were obtained from agarose gel electrophoresis and ethidium bromide staining. **C**, BMDMs were primed with maltol or LPS during the priming step (1st signal) and subjected to maltol or ATP at the activation step (2nd signal), as indicated. The secretion of IL-1β and Casp1 and the expression of NLRP3 were

analyzed by ELISA and immunoblotting. **D**, Mice (n = 5 per group) were injected IP with PBS, maltol (2 mg/mouse), or LPS (100 μg/mouse). After 6h injection, the peritoneal IL-1β secretion was measured using ELISA. The bar graph presents the mean ± SD.

Supplementary Figure S4

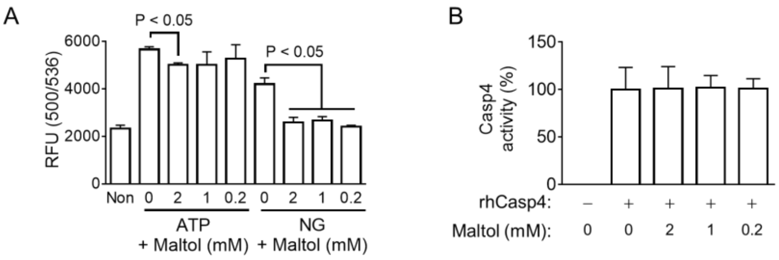

A, LPS-primed BMDMs were treated with DHR123 to measure ROS production in the presence of ATP or NG with/without Maltol. Relative fluorescence unit (RFU) as the level of ROS was measured. **B**, Recombinant human (rh) caspase 4 (Casp4) was incubated with maltol, and the Casp4 activity was analyzed using an assay kit. The bar graph presents the mean ± SD.
